# Reducing Opioid Prescriptions after Common Outpatient Pediatric Urologic Surgeries: A Quality Improvement Assessment

**DOI:** 10.1097/pq9.0000000000000623

**Published:** 2023-01-16

**Authors:** Megan Stout, Seth Alpert, Kelly Kersey, Christina Ching, Daniel Dajusta, Molly Fuchs, Daryl McLeod, Rama Jayanthi

**Affiliations:** From the * Department of Urology, Nationwide Children’s Hospital, Columbus, Ohio; † Department of Quality Improvement Services, Columbus, Ohio.

## Abstract

Pediatric patients undergoing outpatient surgeries often receive prescriptions for postoperative pain, including opioid medications. As a result, the American Academy of Pediatrics formally challenged all pediatric surgeons to decrease opioid prescribing for common specialty-specific outpatient procedures at discharge. To meet this challenge, we designed a quality improvement project to decrease the average number of opioid doses administered to pediatric patients undergoing 3 common outpatient urologic surgeries: circumcision, orchiopexy, and inguinal hernia repair (IHR).

**Methods:**

We formally challenged providers at our institution to reduce opioid doses per prescription and administration to patients overall. We performed a retrospective chart review at our single pediatric institution to establish baseline opioid prescribing values from July 2017 to March 2018. We aimed to reduce this value by 50% in 6 months and sustain this decrease throughout the project duration.

**Results:**

We performed 1,518 orchiopexies, 1,505 circumcisions, and 531 IHRs. The percent change in the average number of opioid doses prescribed per patient from baseline values assessed to 2021 was statistically significant for orchiopexies (*P* < 0.0001), IHRs (*P* < 0.0001), and circumcisions (*P* < 0.0001). In addition, the change in the percentage of patients prescribed opioids from baseline was statistically significant for all 3 procedures (*P* < 0.001).

**Conclusions:**

This project demonstrated that through an organized quality improvement initiative, the average number of opioid medications prescribed and the total percentage of patients prescribed opioids following common outpatient pediatric urologic procedures can be decreased by at least 50% and sustained through project duration.

## INTRODUCTION

The national opioid epidemic remains a critical issue of today’s healthcare system, largely fueled by overprescribing patterns and improper disposal of excess opioid medications. Practitioners are encouraged to carefully consider the need for prescribed narcotics for patients of all ages, including the pediatric population. In March 2018, the American Academy of Pediatrics (AAP) issued a 6-month challenge to surgical providers to select commonly performed procedures and to make concerted efforts to decrease opioid prescribing to pediatric patients at discharge by 50% compared to baseline values.^1,2^

Pediatric patients undergoing outpatient surgeries often receive opioid pain prescriptions for home use that deserve the same monitoring, discretion, and education as their adult counterparts. Ahn et al^3^ reported the results of recent surveys administered to pediatric urologists demonstrating that prescribing opioid medications after most ambulatory surgeries is common despite the belief that patients do not take most of the medication given. Previous studies have also examined overprescribing patterns with common pediatric urologic procedures, reporting that postoperative surveys revealed overall low utilization of the total number of opioid doses provided homegoing.^4,5^ Cardona-Grau et al^6^ found that no significant difference was reported in comparative pain scores even when consciously reducing the number of prescribed doses by 50%. These findings highlight that even in this specific patient population, there is room for improvement in reducing the number of opioid prescription medications administered to pediatric patients after ambulatory urologic surgical procedures. At our institution, we realized that we were prescribing nearly 70%–80% of pediatric patients an average of 11 opioid doses for each common outpatient urologic procedure. We realized we could likely markedly reduce these values based on the current literature of low opioid utilization for this population that could likely be managed with anti-inflammatory medications alone.

Our project aim was to reduce the average number of opioid doses prescribed per patient (regardless of provider or procedure performed), and the total percentage of patients prescribed opioids at discharge for those undergoing circumcision, inguinal hernia repair (IHR), and orchiopexy—3 common ambulatory surgical procedures at our institution—by 50% of the established baseline within 6 months following project initiation with hopes of sustaining this improvement ongoing.

## METHODS

### Context

**Fig. 1.**
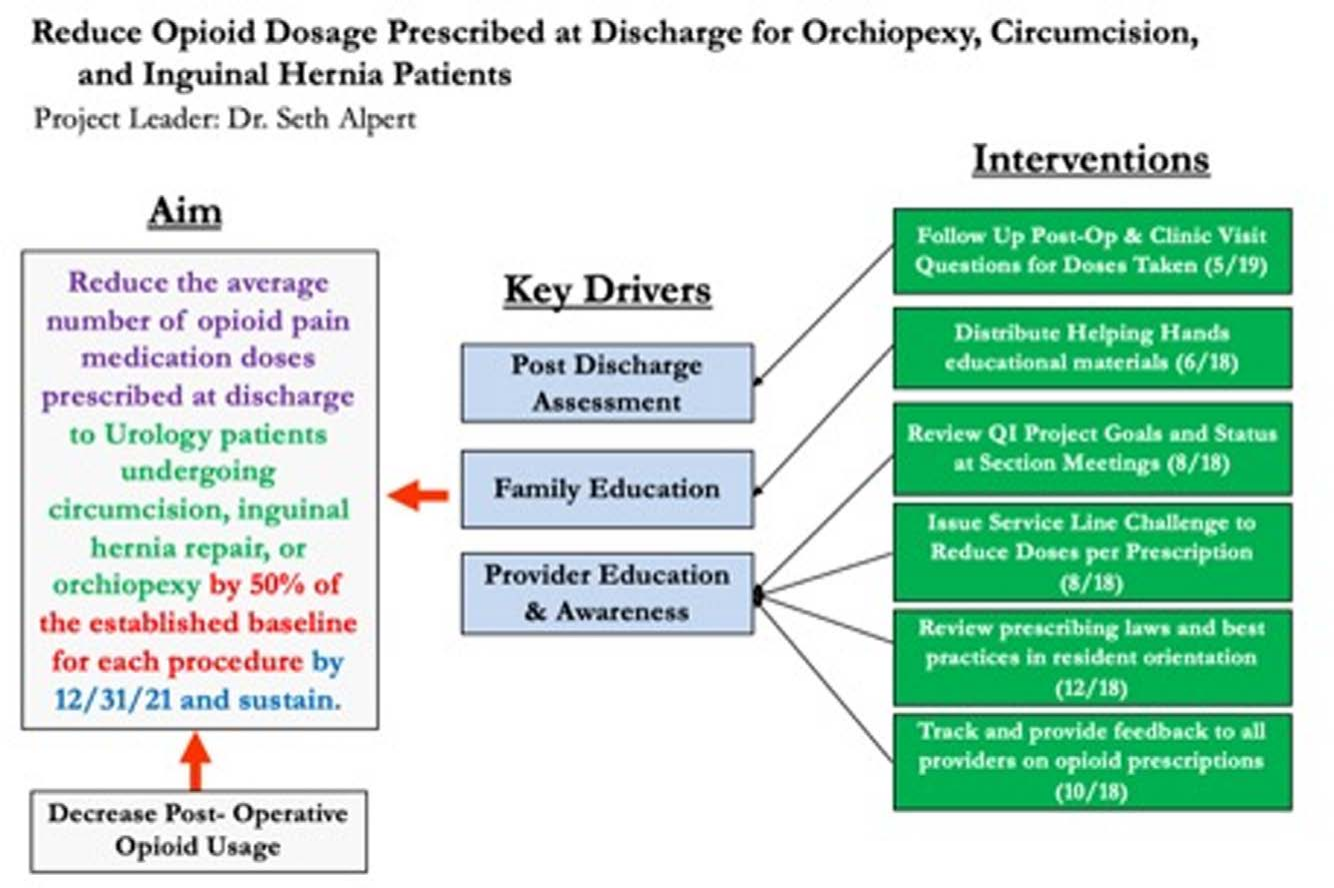
A quality improvement project was initiated for common pediatric urologic procedures—depicted is the overall project aim, key drivers, and timeline of initiatives enacted. Our aim was to reduce the average number of opioid pain medication doses prescribed at discharge to urology patients undergoing orchiopexy, circumcision, and IHR by 50% of the established baseline by December 2018 and sustain this practice pattern at our institution.

Our quality improvement project contextual and key timeline of interventions for development and initiation are outlined in Figure 1. Our key drivers for project organization included improved institutional and urology departmental awareness of the AAP challenge with provider education to encourage and intermittently assess opioid use reduction following the designated pediatric urologic surgical procedures.

## INTERVENTIONS

Institutional review board or quality improvement board approval was not required for this project initiation. This project was initiated at a large, tertiary academic pediatric medical center with a history of an established quality improvement infrastructure making dissemination of new project initiation and guidelines readily feasible. Our institution focuses on disseminating QI knowledge and science, expanding the focus on communication in interdisciplinary health teams and clinical pathway establishment. Within the urology department, our group has an organized quality and safety structure, including monthly project reviews, quality medical directors, scheduled project meetings with project leaders, and quarterly staff meetings. The necessary team for this project and its successful implementation includes a physician leader/project champion, pediatric urologic surgery members, pharmacy, pain management teams, clinical informatics support staff, and clinic nursing staff.

Leading up to project initiation, we formally challenged providers at our institution to reduce opioid doses per prescription and administration to patients overall at a hospital section meeting in July 2018. Since we predominately function at a teaching hospital with residents and advanced practice provider staff that regularly rotate through surgical services, this quality improvement further ensured provider education and awareness by reviewing opioid-prescribing laws and best practices in resident and staff orientation starting in October 2018 via email and in person.

We performed a retrospective chart review at our single pediatric institution to establish the designated baseline opioid prescribing values to reduce this value by 50% in 6 months. In addition, utilizing Qlik Sense (Qlik, King of Prussia, PA) for data ascertainment and organization, we identified CPT codes for orchiopexy (54620, 54640), circumcision (54161), and IHR (49491, 49495, 49500, 49501, 49505, 49520, 49650, 49651) surgical procedures at our institution.

We systematically identified the total number of monthly cases performed, the number of doses in each opioid prescription placed, and the number of patients who were prescribed narcotics following the completion of the surgical procedure within our project period. We collected baseline data from July 2017 to March 2018 and continued data collection through March 2022. Current data collection is presently ongoing. In addition, we tracked and provided feedback to all providers throughout the study duration regarding opioid prescription use at baseline and target goals of reducing use by 50% of the established baseline in 6 months, intending to sustain this decrease throughout the project duration.

## PROCESS MEASURES

Measures chosen for studying processes in place included evaluating the average number of opioid doses prescribed at discharge and the percentage of patients having an opioid prescription upon discharge following each procedure at baseline before any project initiation, education, or intervention.

## OUTCOME MEASURES

This project’s measure of outcome or success of the intervention involves a provider’s process of prescribing opioids after a circumcision, IHR, or orchiopexy procedure, both in the number of total doses prescribed to each individual and in the total number of patients prescribed opioid medication at discharge that was tracked throughout the study.

## BALANCING MEASURES

Balancing measures included any impact from the goal to decrease opioid-prescribing patterns, such as tracking pain control following the procedure. Postoperative phone calls were made from February to October 2019 to assess the patient’s overall status, if school or daycare was resumed, regular bowel movement status, doses taken compared to doses prescribed, how many days doses were needed, if the patient called to request additional opioids, and if there were any parental concerns. Following this period, patient calls for pain management assistance after discharge that resulted in writing additional opioid prescriptions were tracked via electronic medical record documentation. To address pain management by alternative means, we encouraged the use of over-the-counter pain medications with parent directions for the alternation of acetaminophen and ibuprofen at discharge and during return patient phone calls. During the monthly review, provider/prescriber feedback was given throughout the project duration when >10 doses of a prescription were written to refocus the project aim or discuss any limitations to meeting project goals.

## ANALYSIS

We performed descriptive statistics with average doses of opioids prescribed in each annual period for continuous variables, while percentages of patients utilizing opioid scripts were obtained for categorical variables. In addition, we performed unpaired T-test statistical analyses for percent change in the average opioid doses prescribed annually and chi-squared testing to compare the percentage of patients prescribed opioid medications from baseline values to the final year of analysis. Two control charts illustrated data to assist with interval tracking following the interventions initiated. The average opioid doses per prescription by surgical procedure performed were plotted on an x-bar chart. The percent of patients prescribed an opioid at discharge by surgical procedure was plotted on a p-chart. The American Society of Quality rules were applied to detect special cause variation and determine when a shift in performance occurred.^7^

## ETHICAL CONSIDERATIONS

We reviewed the overall ethical aspects of implementing and studying this intervention on pediatric patients, ensuring that the patient’s parent phone calls were returned promptly and that if the pain was inadequately controlled due to our intervention, additional opioid doses were available and administered. There were otherwise no identifiable conflicts of interest.

## RESULTS

**Fig. 2.**
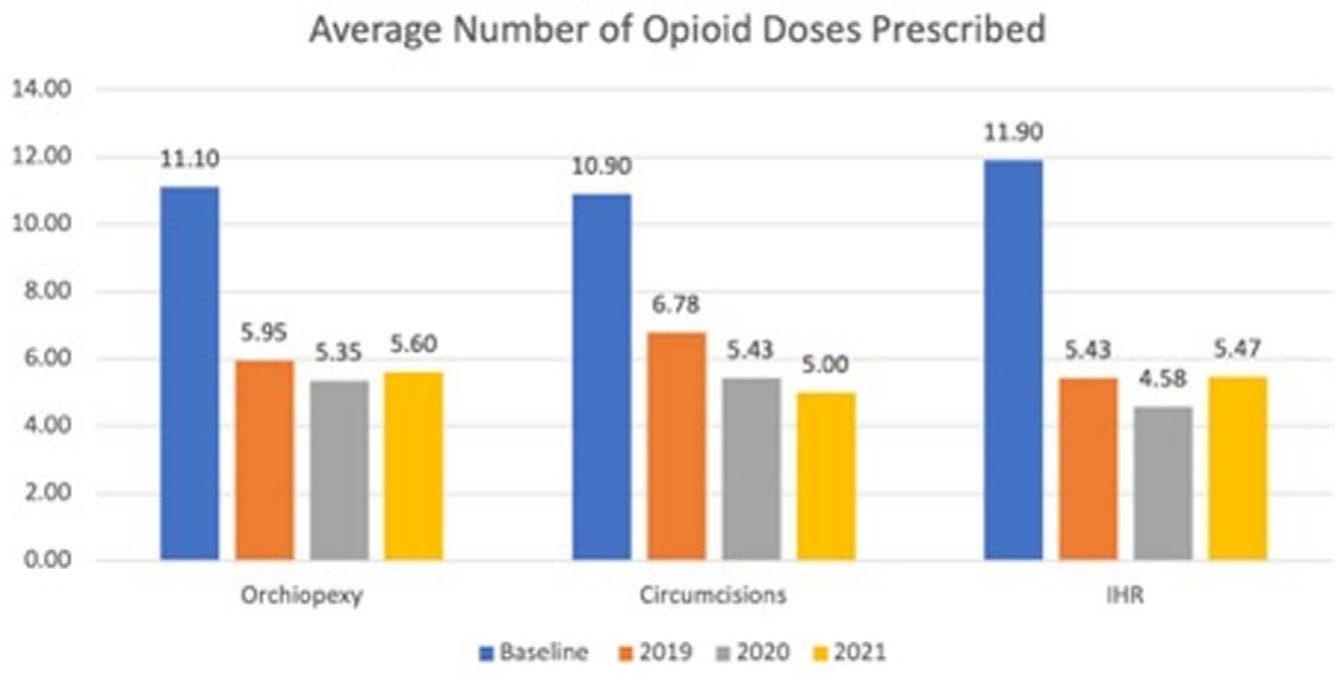
Orchiopexy’s baseline average of 11.1 doses per prescription was decreased to 5.6 doses per script at discharge. For circumcision, a baseline average of 10.9 doses per prescription was decreased to 5 doses at discharge. For IHR, a baseline average of 11.9 doses was decreased to 5.5 at discharge. The percent change of the average number of opioid doses prescribed from baseline values assessed in 2017–2018 to 2021 was statistically significant for orchiopexies (*P* < 0.0001) and IHRs (*P* < 0.0001) and close to statistical significance for circumcisions (*P* = 0.0733).

**Fig. 3.**
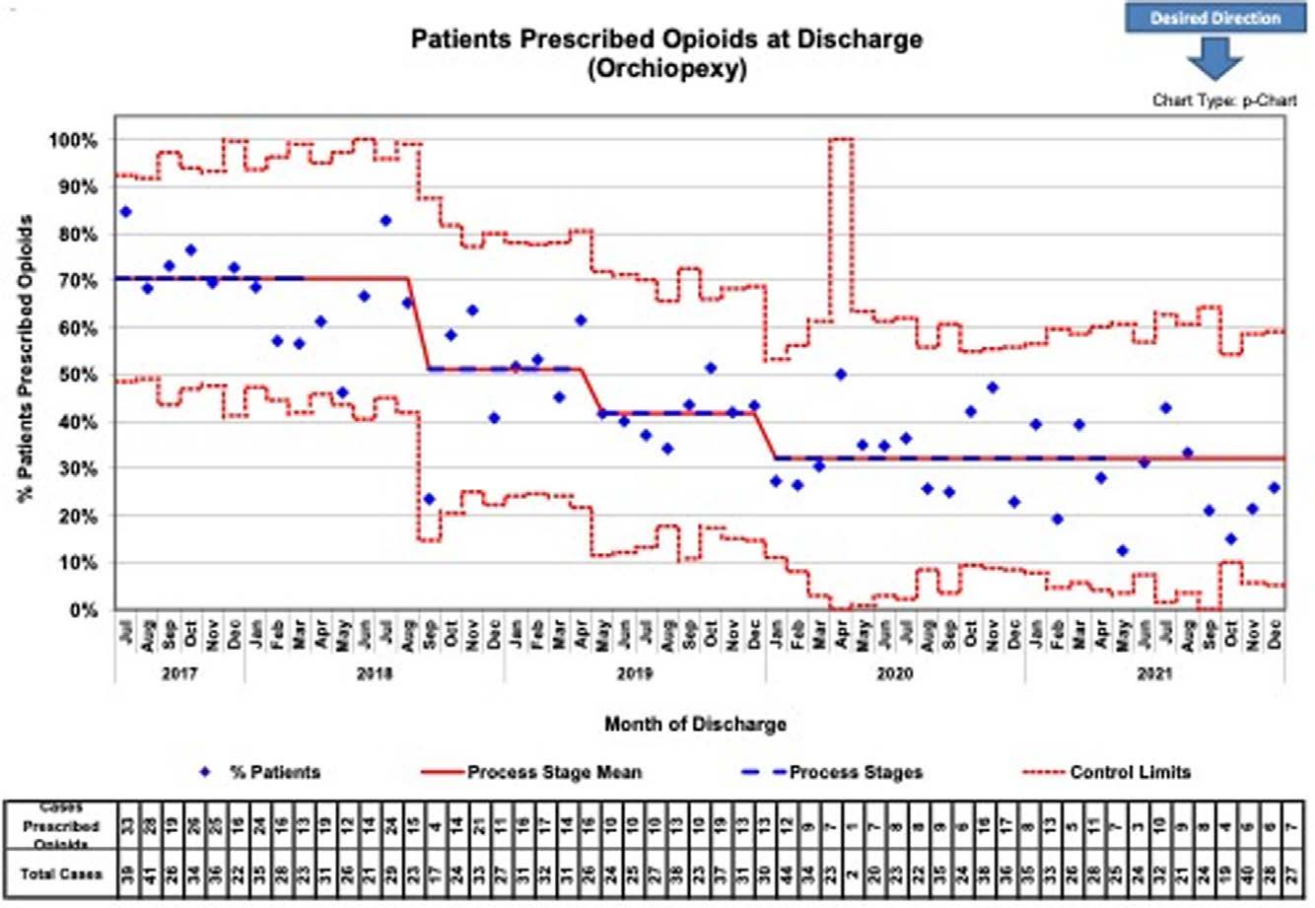
Data were illustrated through control charts. The percent of patients prescribed an opioid at discharge by surgery was plotted on a p-chart. ASQ rules were applied to detect special cause variation and determine when a shift in performance occurred. Baseline data were collected from July 2017 to March 2018. Postoperative phone calls were made from February to October 2019 to assess the patient’s overall status. The annual percentage of orchiopexy patients prescribed opioid medications began at 70% of individuals at baseline and decreased to just 27% at the study’s conclusion. ASQ indicates American Society of Quality.

We performed 1,518 orchiopexies, 1,505 circumcisions, and 531 IHRs during our project. For orchiopexies, the average number of opioid doses prescribed decreased from 11.1 ± 4.5 in 2017–2018 at baseline when the project began to 5.6 ± 2.5 in 2021 (Fig. 2), with the annual percentage of orchiopexy patients prescribed opioid medications starting at 70% at baseline and decreasing to 27% at project conclusion (Fig. 3).

**Fig. 4.**
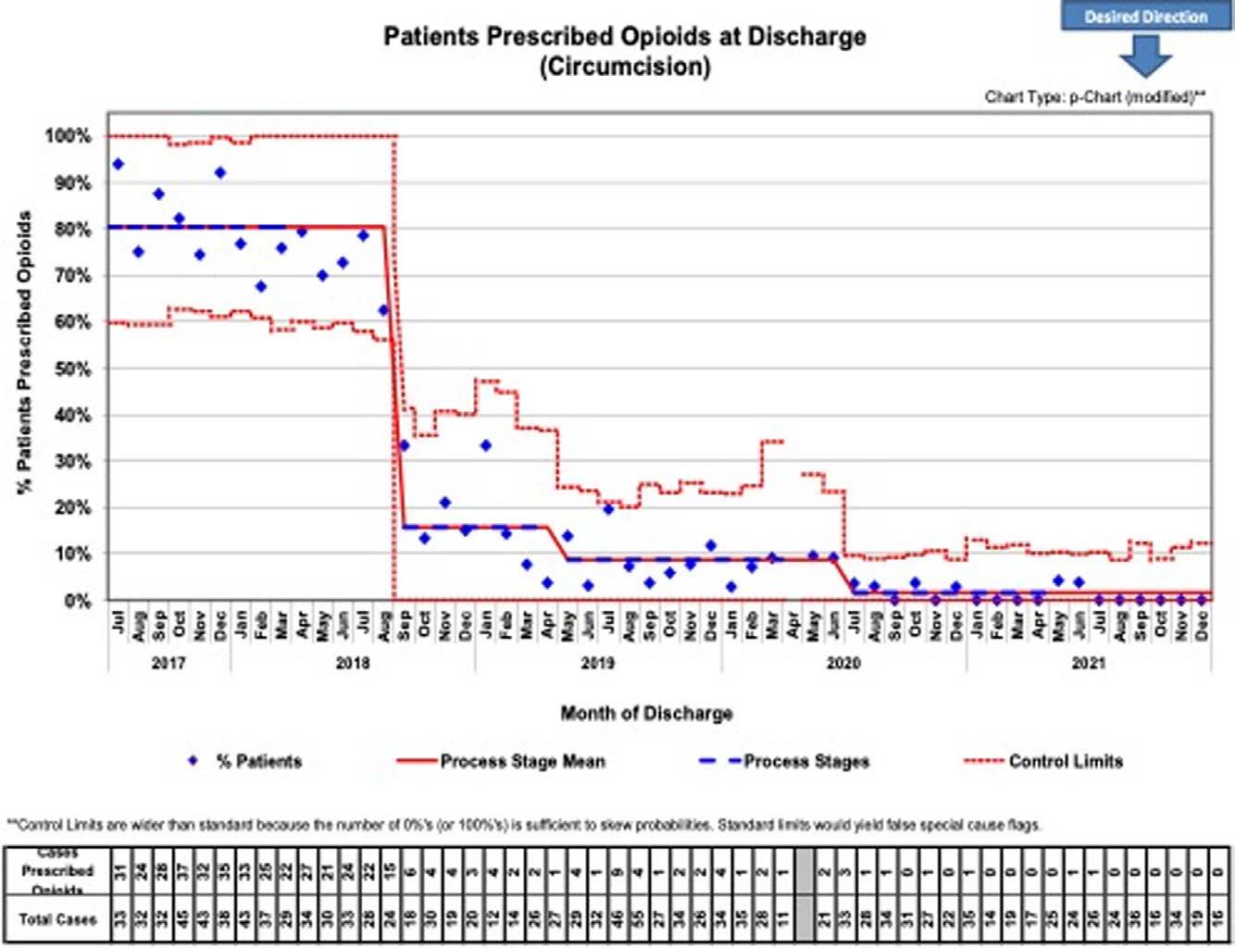
Patients undergoing circumcision began with 81% of individuals being prescribed opioid prescriptions during the baseline period from July 2017 to March 2018, which then declined to just 1% of patients at the quality initiative conclusion.

**Fig. 5.**
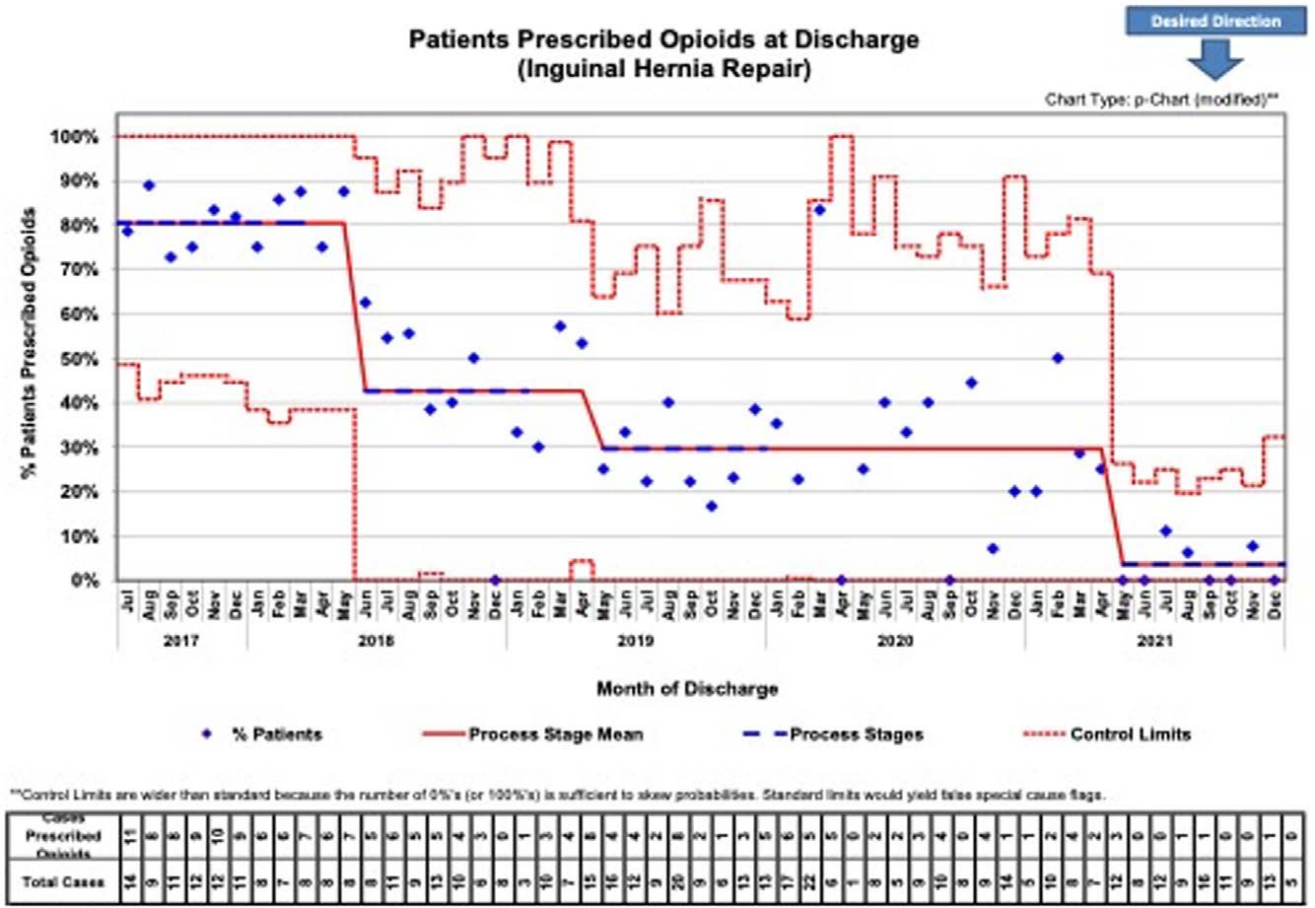
The percentage of patients prescribed opioid medications following IHR went from 80% at baseline (July 2017 to March 2018) to just 15% by 2021.

Circumcisions had a 10.9 ± 0.5 average number of opioid doses ordered at baseline, with 5 ± 0.0 doses noted at the project conclusion, with the percentage of patients prescribed narcotics decreasing from 81% to 1% (Fig. 4). Finally, for IHRs, 11.9 ± 2.5 average opioid doses per script were administered at baseline down to 5.5 ± 0.9 at project completion, with the total percentage of patients receiving medications decreasing from 80% to 15% by 2021 (Fig. 5). As depicted through our control charts with changes in percentages of patients prescribed opioid doses over time, there are clear process changes that occurred due to the interventions implemented. These changes were sustained over time (as was the initial project aim) with all 3 procedures.

Within 6 months of project initiation, process measures were evaluated. The average number of doses prescribed and the percentage of patients receiving narcotics decreased to 50% or less of baseline values. We could sustain this result for most of the project duration with all 3 procedures. The percent change of the average number of opioid doses prescribed from baseline values assessed in 2017‐2018 to 2021 was statistically significant for orchiopexies (*P* < 0.001), IHRs (*P* < 0.001), and circumcisions (*P* < 0.001). In addition, the change in the percentage of patients overall prescribed opioids from baseline compared to the year of project completion was statistically significant for all 3 procedures (*P* < 0.001).

Based on a review of patient-parent phone calls as a balancing measure, only 2 orchiopexy patients, 1 circumcision patient, and no IHR patients stated that pain was uncontrolled by the alternation of medications such as ibuprofen or acetaminophen alone (N = 3). Of all patients prescribed opioid prescriptions (N = 21/54, 38%) following their procedure throughout the project duration, 48% of the patients with doses prescribed did not take any of the doses at all, 1 patient took all of the prescribed doses, and only 1 patient-parent phone call was performed stating that pain control remained inadequate. This individual was post-circumcision and included 1 of the 3 additional parent phone calls throughout the project duration—by 5 days post-procedure, this individual had improvement in pain control on the second follow-up phone call; therefore, no additional narcotic medications were prescribed. There were no reported concerns from other ancillary operative teams, such as anesthesiology, regarding the undertreatment of pain throughout the project.

## DISCUSSION

The opioid epidemic has a critical impact on our healthcare system, even at the pediatric patient level of care. Pediatric patients undergoing surgical intervention receive narcotic scripts commonly. Therefore, continued institutional and provider education and monitoring of prescribing habits and patterns are imperative. The AAP challenged surgical providers to target commonly performed pediatric procedures and to make concerted efforts to decrease opioid prescribing to patients in practice.^1,2^ At our institution, after formally announcing the AAP challenge to surgical providers, we evaluated our baseline prescribing patterns following common pediatric urologic ambulatory procedures, including circumcision, IHR, and orchiopexy, and continued monitoring over several years after initiatives occurred to reduce opioid prescription use. We found that within 6 months of project initiation, the average number of doses prescribed and the percentage of patients receiving narcotics decreased to 50% or less of baseline values. This result was sustainable for the project duration for all 3 procedures. In addition, the decrease in the percentage of patients prescribed opioids from baseline compared to end project data in 2022 was statistically significant for all 3 procedures evaluated, with only 1 patient-parent phone call in the postoperative period requesting additional opioid scripts for persistent pain.

Prescribing patterns of opioid medications following pediatric urologic procedures and their widespread use for postoperative pain control have previously been described. Bilgutay et al^4^ (2020) reviewed 202 patients undergoing penile, scrotal, and inguinal surgical interventions. They found that most patients only used 0‐2 doses of prescription pain medication after discharge following outpatient urologic surgery. Hunsberger et al^5^ (2020) interviewed 115 patients following circumcision or orchiopexy procedures, with the majority of patients reporting that the median number of tablets prescribed was 30, the median reported number used was 4 for 2 days, with 75% of opioids prescribed not consumed. Similarly, in our study, nearly 50% of patients prescribed opioid doses following outpatient urologic procedures did not use a dose, while only 1 patient assessed had utilized all doses prescribed. Studies demonstrating the significant disproportion of doses prescribed and the number utilized by patients show the lack of awareness of provider prescribing patterns and actual patient-perceived pain needs following common urologic procedures. Patient-parent questionnaires demonstrated no significant difference in pain scores when doses prescribed were reduced.^6,8^

In 2019, Ahn et al^3^ administered a survey to pediatric urologists evaluating typical baseline prescribing patterns—of the 102 respondents, nearly half of providers reported prescribing narcotics postoperatively for all pediatric urologic routine procedures. However, most did so despite believing that patients do not take most of their prescribed medication. Several recent articles highlight the utility of analyzing opioid prescription patterns through methods such as clinical practice guidelines and considering alternative opioids to reduce opioid misuse.^9^ Quality initiative protocols and government-mandated consents for pediatric patients demonstrate that possible standardization and structural organization can lead to fewer patients receiving opioid prescriptions after minor or outpatient procedures and a significant and sustained drop in opioid medications.^10,11^ Examples of similar quality improvement projects organized in other subspecialty fields have had similar aims and efficacy of decreased opioid medication consumption.^12–15^

Utilizing these principles after deciding to proceed with the AAP’s challenge statement, we reviewed our department’s baseline dose administration for common urologic outpatient procedures (circumcision, orchiopexy, and IHR) before the initiation of our project. We also reviewed our state’s prescribing laws to inform all levels of prescribing providers (surgeon, resident physician, and advanced practice provider) and any changes that may affect our prescribing patterns. For example, we found that new limits on prescription opioid use for acute pain in 2017 in our state allotted no more than 5 days of opioids that can be prescribed to minors on the initial script and only after written consent from a parent or guardian is obtained. Otherwise, healthcare prescribers may prescribe over this limit only after a specific clinical reason is stated in the patient’s record. In addition, in June 2018, state law required all CPT codes for each procedure to be linked to the individual opioid script written. Our state also released readily available prescriber resources such as a morphine equivalent dose calculator, conversion tables, and general prescriber resources. These regulations and new prescribing patterns were well received by our surgery team, rotating resident physicians, and advanced provider staff, with no major difficulties regarding following these regulations when prescribing.

Through the announcement to the urology department and support staff of our acceptance of the service line challenge to decrease opioid prescribed doses by 50% and through the implementation of our systemic processes with balancing measure assessments, we saw a significant decrease in the average number of doses and percentage of patients prescribed in all 3 procedures in the following months. These results are similar to other institutions in which heightened awareness alone of the number of doses administered had greatly reduced the amount prescribed in a matter of years.^16^

Although our project highlights improving opioid prescribing patterns by significantly and sustainably reducing the average number of doses prescribed and the percentage of patients prescribed narcotic medications, it is not without limitations. We established a relative baseline in 2017‐2018 for opioid prescribing data patterns for circumcision, IHR, and orchiopexy; however, we did not pursue data for other common outpatient urologic surgical procedures (ie, hypospadias repair) to evaluate the baseline prescribing trends. Although we followed a subset of patients with follow-up phone calls to assess overall pain control and opioid use and documented parent-patient phone call encounters following the intervention, which explicitly requested more opioid scripts, we did not obtain subjective survey data for all patients. There was no standardization between providers on prescribing dosage patterns beyond increased awareness at project initiation and education of support staff regarding project implementation and best prescribing practices, which may reflect why there are deviations in doses of opioids prescribed within each surgical procedure. We also did not analyze doses prescribed between various age groups. This fact may be reflected by the minimal doses of narcotics prescribed within the circumcision group since this procedure is likely arbitrarily performed on an overall younger cohort of patients. Finally, this is a single institution quality improvement project for a pediatric urologic patient population, which may present data not generalizable to other institutions with different practice patterns or without a strong foundational quality improvement infrastructure already organized.

Our project initiative emphasizes that there should be institutional assessment and provider awareness of the number of opioid prescriptions given following common pediatric urologic ambulatory procedures. Through acceptance of a quality improvement challenge, conscious reduction of average doses and patients prescribed by 50%, and continued education of providers’ overall prescribing patterns, we found that opioid scripts can be greatly reduced and sustained over several years of practice. Future directions of study include continued longitudinal data collection at our own tertiary pediatric medical center with the implementation of standardized education and protocols for opioid-prescribing practices for each procedure. Similar reforms can also be organized at the multi-institutional level with a call to answer the AAP challenge, obtain baseline prescribing pattern data, and project initiation when all prescribing providers know the goals of decreasing opioid doses prescribed. Our quality improvement initiative is just one critical step to decreasing narcotic use and abuse in the pediatric health system.

## CONCLUSIONS

The opioid epidemic is prevalent in today’s healthcare system, affecting adult and pediatric patients. To make quality improvement efforts at the institutional level, it is clear that interventions can begin with evaluation and awareness of opioid-prescribing patterns for common procedures at baseline. This project demonstrated that through an organized quality improvement initiative, the average number of opioid medications prescribed and the total percentage of patients prescribed following common outpatient pediatric urologic procedures can be decreased by at least 50% and sustained through project duration. This project also demonstrates that most pediatric urology patients undergoing circumcision, orchiopexy, or IHR can likely have their pain adequately addressed with over-the-counter medications alone, with the majority of patients by project end requiring minimal to no opioid prescriptions. Of the 21 patients who were prescribed opioid medications (n = 21/54, 38%), only 1 parent called in return due to inadequate pain control with the dosage of opioid medication prescribed following project intervention. Continued education of prescribing providers, support staff, and institutional awareness can reduce opioid prescription utilization for most common pediatric urologic outpatient procedures, which warrants future longitudinal project implementations at similar institutions.

## DISCLOSURE

The authors have no financial interest to declare in relation to the content of this article.
